# Brown Fat Expresses Adiponectin in Humans

**DOI:** 10.1155/2013/126751

**Published:** 2013-11-14

**Authors:** Gianluca Iacobellis, Cira Di Gioia, Luigi Petramala, Caterina Chiappetta, Valentina Serra, Laura Zinnamosca, Cristiano Marinelli, Antonio Ciardi, Giorgio De Toma, Claudio Letizia

**Affiliations:** ^1^Division of Diabetes, Endocrinology and Metabolism, Department of Medicine, Miller School of Medicine, University of Miami, Miami, FL 33136, USA; ^2^Department of Radiological Sciences, Oncology and Anatomical Pathology, Sapienza University of Rome, 00165 Rome, Italy; ^3^Department of Internal Medicine and Medical Specialties, Sapienza University of Rome, 155 Viale del Policlinico, 00165 Rome, Italy; ^4^Department of Surgery “P. Valdoni”, Sapienza University of Rome, 00165 Rome, Italy

## Abstract

The presence of brown adipose tissue (BAT) in humans is unclear. Pheochromocytomas (PHEO) are rare tumors of neuroectodermal origin which occur in 0.1-0.2% of patients with hypertension. We sought to evaluate the presence and activity of BAT surrounding adrenal PHEO in a well-studied sample of 11 patients who were diagnosed with PHEO and then underwent adrenalectomy. Areas of white fat (WAT) and BAT surrounding PHEO were obtained by Laser Capture Microdissection for analysis of uncoupling protein (UCP)-1 and adiponectin mRNA expression. Adiponectin and UCP-1 mRNA levels were significantly higher in BAT than in WAT (0.62 versus 0.15 and 362.4 versus 22.1, resp., *P* < 0.01 for both). Adiponectin mRNA levels significantly correlated with urinary metanephrines (*r* = 0.76, *P* < 0.01), vanilly mandelic acid (VMA) (*r* = 0.95, *P* < 0.01), and serum adiponectin levels (*r* = 0.95, *P* < 0.01). Serum adiponectin levels significantly decreased (24.2 ± 2 **μ**g/mL versus 18 ± 11 **μ**g/mL, *P* < 0.01) after adrenalectomy in PHEO subjects. This study provides the following findings: (1) BAT surrounding PHEO expresses adiponectin and UCP-1 mRNA, (2) expression of adiponectin mRNA is significantly higher in BAT than in WAT surrounding PHEO, and (3) catecholamines and serum adiponectin levels significantly correlate with BAT UCP-1 and adiponectin mRNA.

## 1. Introduction

The role and presence of brown adipose tissue (BAT) in humans is intriguing, but far to be clearly understood. BAT in humans might play an important role in thermogenesis and possibly energy expenditure [1]. Recent findings suggest that BAT may be more prevalent and active in adult humans than ever thought [2].

BAT is normally present in the human fetus, gradually decreasing over the first decade of life [3]. Remnants of BAT in adults are usually found in the neck, mediastinum, axilla, retroperitoneum, paravertebral regions, and abdominal wall [3].

Active BAT was firstly described through ^18^F-FDG positron emission tomography (PET) in patients affected by adrenal phaeochromocytoma (PHEO) as intense uptake by BAT reduced after removal of PHEO [4, 5] Thus, PHEO may represent a unique model *in vivo* for the evaluation of BAT in subjects exposed to high levels of catecholamine, which represent important regulator of BAT.

PHEO are rare tumors of neuroectodermal origin which occur in 0.1-0.2% of patients with hypertension [6]. Whether BAT surrounding PHEO is not an innocent bystander, but rather an active player in controlling metabolic changes and energy expenditure, has been suggested, but not fully elucidated [7]. Whether BAT in general is also a source of metabolically active adipokines, such as adiponectin, is unclear. Moreover, adiponectin has been suggested to play a role in PHEO, although data are controversial [8]. 

Our hypothesis was to evaluate the presence and endocrine activity of BAT in adipose tissue surrounding adrenal PHEO as specimen of visceral adipose tissue in a model of chronic hypercatecholaminism, in a well-studied sample of patients who were diagnosed with PHEO and then underwent adrenalectomy. Areas of both white fat (WAT) and BAT surrounding the tumor were obtained by Laser Capture Microdissection (LCM) for selective TaqMan real-time quantitative PCR analysis of uncoupling protein (UCP)-1, specific marker of BAT, and adiponectin mRNA expression [9, 10]. 

## 2. Materials and Methods

### 2.1. Subjects

Eleven consecutive patients with PHEO (5 males, 6 females; mean age 52 ± 15 yrs) who were referred to our Day Hospital for secondary hypertension were studied. The diagnosis of PHEO was made on the basis of (1) clinical symptoms (palpitations, headache, diaphoresis, and hypertension); (2) elevated values of 24-hour urinary metanephrines and vanillymandelic acid (VMA) [5]. PHEO was radiologically evident at the computed tomography (CT), magnetic resonance imaging (MRI), or scintigraphy in each patient. The diagnosis was histologically confirmed after surgical removal of the tumor. Twenty normotensive, nondiabetic, healthy subjects were recruited to form a control group for baseline comparison. Clinical postsurgery assessment was performed after 6 months in all PHEO subjects.

The study was approved by the Human Ethical Committed Department of Internal Medicine and Medical Specialties, University of “Sapienza”, Rome, Italy, and conducted with the principles of the declaration of Helsinki. 

### 2.2. Clinical and Laboratory Data

Office blood pressure (BP) was measured with a standard mercury manometer with the subjects sitting for 5 minutes; hypertension was defined by BP measurement of systolic (S) (SBP) >140 and diastolic (D) (DBP) >90 mmHg. After an overnight fast, blood samples were obtained in all study subjects. Twenty-four-hour total urinary metanephrines were measured by RIA method (normal range 30–350 *μ*g/24 h) and VMA (normal range 1–10 mg/24 h).

### 2.3. Surgical Treatment

During the preparation for surgical treatment, doxazosin (dose between 4–12 mg/day), which blocks *α*1-adrenergic receptors, was administered. Pharmacotherapy was administered for 2 weeks before scheduled surgical treatment. Administration of *β*-receptor blockers (atenolol at the dose between 50 and 100 mg/day) was performed in patients with reflex tachycardia or extrasystolic arrhythmia. The drugs were in all patients well tolerated; no orthostatic hypotension was observed. All patients received preoperative volume therapy with intravenously sodium intake. The patients underwent adrenalectomy at the Department of Surgery “Pietro Valdoni,” and a laparoscopic adrenalectomy was performed in all PHEO patients.

### 2.4. Serum Adiponectin Assay

Serum adiponectin levels were measured at diagnosis and 6 months after adrenalectomy. Samples of venous blood obtained were immediately centrifuged and the serum was stored at −80°C, until assayed. Adiponectin levels were measured by using the Human Adiponectin ELISA Kit (BIO Ventor, Laboratory of Medicine, Candler, NC). Intra- and interassay coefficients of variation for adiponectin were 6.2% and 7.2%, respectively.

### 2.5. Adiponectin and UCP-1 mRNA

Formalin-fixed and paraffin-embedded tumor specimen was obtained from four PHEO patients. Hematoxylin and eosin (H&E) stained sections were performed for histological evaluation. In each sample of pheochromocytoma we found surrounding WAT with focal areas of BAT. Areas of both WAT and BAT were obtained by laser capture microdissection (LCM) for selective analysis of adiponectin and UCP-1 mRNA expression [11, 12]. For microdissection by Leica LMD 7000 (Leica Microsystems, MI, Italy), 5 um thick sections were performed and stained with hematoxylin and eosin. Total RNA from microdissected tissue was extracted by using High Pure FFPE RNA Micro Kit (Roche Diagnostics, Indianapolis, IN, USA) according to the supplier's instructions, which provided an elution in a final volume of 20 *μ*L. The RNA was stored at −20°C until it was used. Then, total RNA was reverse-transcribed in a final volume of 20 *μ*L using High Capacity cDNA Reverse Transcription Kit (Applied Biosystems, Foster City, CA, USA).

TaqMan real-time quantitative PCR for adiponectin and UCP-1 mRNA was performed on an ABI PRISM 7500 Fast Real-Time PCR System (applied biosystems, Foster City, CA, USA). We evaluated the expression of adiponectin and UCP-1 between homogeneous population of brown and white adipocytes compared to the housekeeping gene GAPDH; thus, values were expressed as “relative quantity” (RQ). PCR products for Adiponectin and UCP-1 were detected using gene-specific primers and probes labeled with reporter day FAM (adiponectin gene ID: 9370, UCP-1 gene ID: 7350, Applied Biosystems, Foster City, CA, USA). GAPDH was detected using gene-specific primers and probes labeled with reporter day FAM (gene ID: 2597, Applied Biosystems, Foster City, CA, USA). Amplification product length of these assays was 71, 68, and 93 base pairs, respectively. PCR reaction was carried out in triplicate on 96-well plate with 20 *μ*L per well using 1X TaqMan Master Mix. After an incubation for 2 minutes at 50°C and 10 minutes at 95°C, the reactions continue for 40 cycles at 95°C for 15 seconds and 60°C for 1 minute. At the end of the reaction, the results were evaluated using the ABI PRISM 7500 software. The Ct (cycle threshold) values for each set of three reactions were averaged for all subsequent calculations. 

### 2.6. Statistical Analysis

Statistical analysis was performed by using Sigmastat (Jandel Corporation). All data were expressed as mean ± standard deviation (±SD). Comparison between parameters was calculated by paired and unpaired *t*-test. Correlations between variables were assessed by simple linear regression analysis. *P* value < 0.05 was considered statistically significant.

## 3. Results 

### 3.1. Baseline

As expected, fasting blood glucose, office blood pressure (systolic and diastolic values), and heart rate were significantly higher in PHEO patients than in controls. No differences in age, BMI, waist circumference (WC), creatinine, lipid profile, and serum adiponectin were found between PHEO patients and controls (Table 1). At baseline, we found a positive correlation between serum adiponectin and VMA values (*r* = 0.60, *P* < 0.01) in all patients with PHEO.

### 3.2. Postsurgery Outcomes

PHEO was benign in 10 patients (90%), with a mean diameter of the tumor at 4 cm (range 1–10 cm), whereas a malignant form was detected in 1 patient (10%, mean tumor diameter of 11 cm). Blood pressure, urinary metanephrines, and VMA levels significantly decreased (Table 2), as well as reduction of fasting blood glucose (102 ± 15 mg/dL versus 84 ± 8 mg/dL; *P* < 0.001) and serum adiponectin levels (24.2 ± 2 *μ*g/mL versus 18 ± 11 *μ*g/mL, *P* < 0.01) after adrenalectomy in PHEO subjects.

### 3.3. BAT and WAT Adiponectin and UCP-1 mRNA Expression

**Figure 1:**
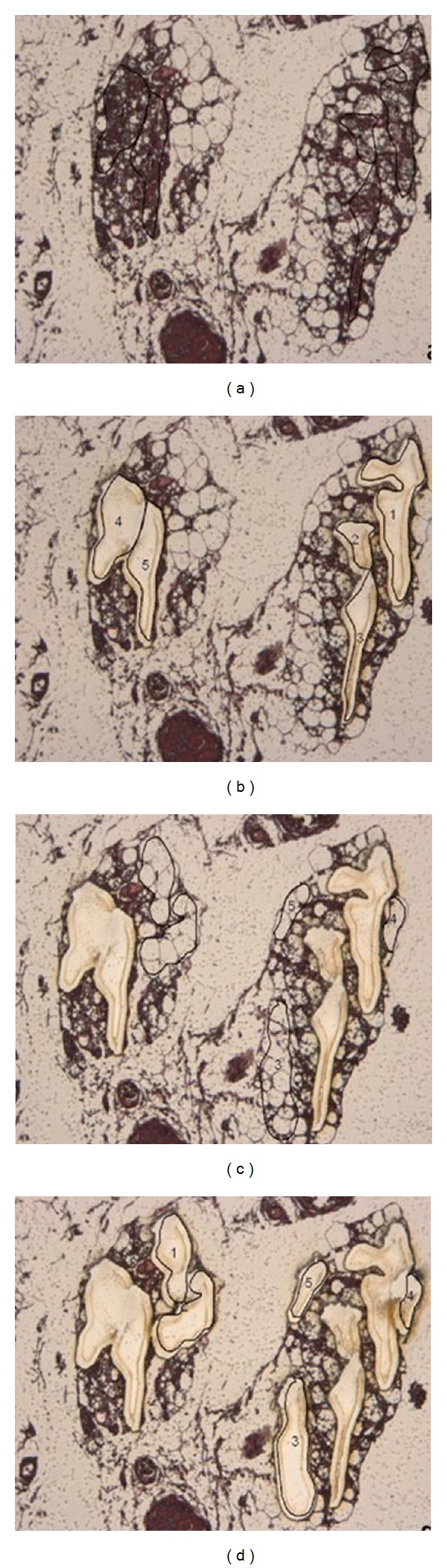
Images from LCM performed on a representative H&E stained adipose tissue section, 20x (a) selection of brown adipose tissue, characterized by multilocular morphology, surrounded by black lines; (b) microdissection and isolation of four areas [1–4] of brown adipose tissue; (c) selection of white adipose tissue, characterized by monovascular cells, surrounded by black lines; (d) microdissection and isolation of white adipose tissue [1–5].

**Figure 2:**
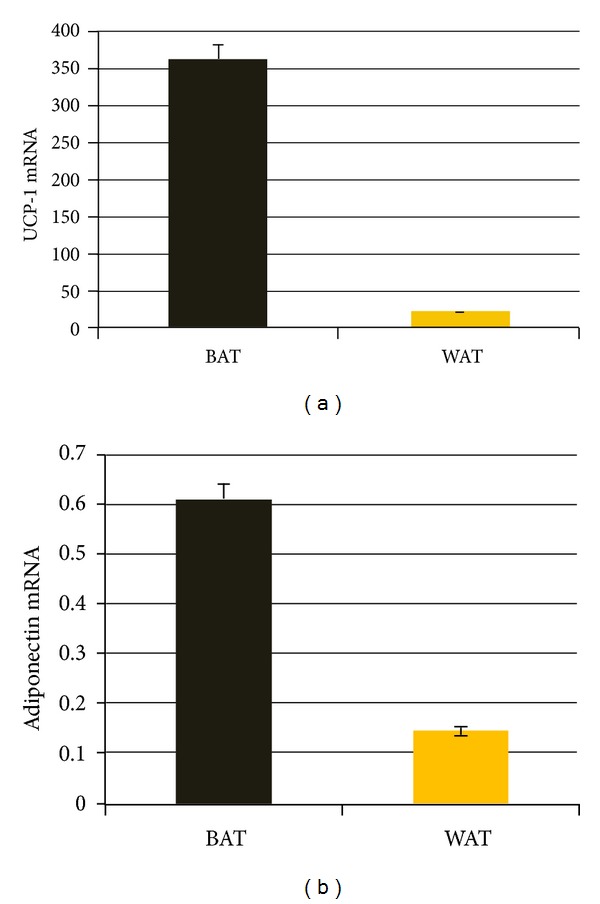
UCP-1 (a) and adiponectin (b) mRNA levels were higher in brown adipose tissue (BAT) than in white adipose tissue (WAT) surrounding the PHEO (values expressed as RQ (relative quantity) (0.62 versus 0.15 RQ; 362.4 versus 221 RQ, resp., *P* < 001 for both).

Although attention was paid to BAT presence during each surgery, BAT surrounding PHEO was identified in four patients. LCM allowed us to select a homogeneous population of BAT and WAT from specific histological areas of PHEO specimens (Figure 1). We analyzed adiponectin and UCP-1 mRNA expression from BAT and WAT obtained from these four patients. Adiponectin and UCP-1 mRNA levels were significantly higher in BAT than in WAT (0.62 versus 0.15 RQ and 362.4 versus 22.1 RQ, resp., *P* < 0.01 for both) (Figure 2). 

Notably, urinary metanephrines were significantly higher (405 ± 32 versus 390 ± 28 *μ*g/24 hours, *P* < 0.05) in those patients presenting BAT surrounding the tumor. 

Linear regression analysis showed that BAT adiponectin mRNA levels significantly correlated with presurgical urinary metanephrines (*r* = 0.76, *P* < 0.01), VMA (*r* = 0.95, *P* < 0.01), and serum adiponectin levels (*r* = 0.97, *P* < 0.01) but not with BMI.

A different linear regression analysis demonstrated that BAT UCP-1 mRNA levels significantly correlated with pre-surgical urinary metanephrines (*r* = 0.98, *P* < 0.01), VMA (*r* = 0.93, *P* < 0.01) but not with BMI.

## 4. Discussion

This study provides the following findings: (1) human BAT expresses adiponectin, (2) expression of adiponectin mRNA is significantly higher in BAT than in WAT surrounding PHEO, and (3) urinary metanephrines and adiponectin levels significantly correlate with BAT UCP1 and adiponectin mRNA levels. This is the first time that human adult BAT is found to express an adipose tissue-specific cytokine such as adiponectin. Achieving the knowledge that human BAT expresses adipokines with metabolic and hemodynamic properties may be of great relevance. Regulation of adiponectin expression in human and animal WAT has been well studied, whereas there is no data on the expression of adiponectin in human BAT. For example, in adipocytes (WAT) from mice model, acute catecholamine administration is associated with development of insulin resistance due to reduced plasma levels of adiponectin and increased mRNA levels of receptors of adiponectin (adipoR2) [13–15].

Previously, adiponectin expression from brown adipocyte cell line T37i was described in experimental animal models [16, 17]. Fujimoto et al. reported in the animal fetus that adiponectin is synthesized in BAT and surrounding immature tissues and then secreted into the blood [18]. Interestingly, adiponectin and adiponectin receptors (Adp R1 and R2) gene expressions have been found in pheochromocytoma tissue and associated with the level of catecholamine produced in the tumor [19]. Based on our results, it is possible to speculate that chronic elevated levels of catecholamines may contribute to BAT adiponectin secretion. Interestingly, the significant postsurgical decrease in serum adiponectin levels was associated with the surgical removal of catecholamine-induced activation of BAT. 

BAT presence in PHEO has been previously detected, but with uncertain role. PHEO is typically characterized by overproduction of catecholamines (epinephrine and norepinephrine), which can interact with BAT through the stimulation of catecholamine receptors [19]. Our data may support the hypothesis that BAT activity in adult humans can be activated by elevated plasma catecholamines such as in the case of patients with PHEO. It could be plausible to hypothesize that the weight loss, commonly observed in patients with PHEO, may be attributed to the catecholamine-induced activation of BAT and increased energy expenditure. It would be certainly of interest to explore whether this speculated weight loss mechanism occurs during weight loss interventions in a more general obese population. However it is more likely that the combinatory effect of multiple hormonal and hemodynamic factors can explain the weight loss. 

Although same findings of this study are interesting, we acknowledge the limitation that, given the nature of the study, they have been generated from a small sample size and therefore no pathophysiological conclusions can be drawn. Moreover, the potential metabolic role of BAT surrounding PHEO that could be generalized to BAT is unknown. The effect of therapeutic alpha blockade on adiponectin expression and plasma concentration could be also considered. However, we believe that our data may open new interesting avenues in the pathophysiology of BAT and its clinical implication in obesity. 

## Figures and Tables

**Table:** (a)

	Age (years)	Sex (m/f)	BMI (Kg/m^2^)	WC (cm)	SBP (mmHg)	DBP (mmHg)	HR (bpm)
PHEO n.11	55.6 ± 14	5/6	23.6 ± 3	88 ± 9	135 ± 10*	82 ± 8*	78 ± 12*
NS n.20	55.7 ± 6	11/9	23 ± 1.4	81.4 ± 2.8	115 ± 4	74 ± 5	75 ± 6
*P*	—	—	—	—	*P* < 0.001	*P* < 0.001	*P* < 0.001

**Table:** (b)

	Fasting glucose (mg/dL)	Creatinine (mg/dL)	Total cholesterol (mg/dL)	LDL (mg/dL)	HDL (mg/dL)	Triglycerides (mg/dL)	Adiponectin (*µ*g/mL)	Urinary metanephrines (30–350 *μ*g/24 h)	Vanillylmandelic acid (1–10 mg/24 h)
PHEO n.11	102 ± 15*	0.9 ± 0.11	197 ± 29	114 ± 29	59 ± 11	118 ± 29	24.2 ± 16	399 ± 30*	15 ± 3.2*
NS n.20	84 ± 8	0.9 ± 0.2	192 ± 30	116 ± 35	57 ± 14	95 ± 32	22.4 ± 8	85 ± 12	7 ± 2
*P*	*P* < 0.001	—	—	—	—	—	—	*P* < 0.001	*P* < 0.001

**Table 2:** Anthropometric, clinical, and biochemical parameters in NF1 patients before and after surgical intervention (adrenalectomy).

	BMI (Kg/m^2^)	WC (cm)	SBP (mmHg)	DBP (mmHg)	HR (bpm)	Fasting glucose (mg/dL)	Adiponectin (*μ*g/mL)	Urinary metanephrines (30–350 *μ*g/24 h)	Vanillylmandelic acid (1–10 mg/24 h)
PHEO Pre n.11	23.6 ± 3	88 ± 9	135 ± 10*	82 ± 8*	78 ± 12	102 ± 15*	24.2 ± 16°	399 ± 30*	15 ± 3.2*
PHEO Post n.11	24 ± 3	89 ± 8	120 ± 8	73 ± 8	82 ± 10	84 ± 8	18 ± 11	85 ± 12	7 ± 2
*P*	—	—	*P* < 0.001	*P* < 0.001	—	*P* < 0.001	*P* < 0.01	*P* < 0.001	*P* < 0.001
